# Rapid Detection of Pesticide Residues in Leaf Vegetables by SERS Technology

**DOI:** 10.3390/s25164912

**Published:** 2025-08-08

**Authors:** Fang Peng, Shuanggen Huang, Qi Chen, Ni Tong, Yan Wu

**Affiliations:** 1 School of Computer Science & Engineering, Jiangxi Agricultural University, Nanchang 330045, China; peng5181@jxau.edu.cn (F.P.); chenqi567726@jxau.edu.cn (Q.C.); 2 Key Laboratory of Modern Agricultural Equipment, Jiangxi Agricultural University, Nanchang 330045, China; shuanggenhuang1979@jxau.edu.cn (S.H.); tongn@stu.jxau.edu.cn (N.T.)

**Keywords:** leafy vegetable pesticide, SERS, AuNPs, DFT, rapid detection

## Abstract

Organophosphate pesticides, fungicides, and neonicotinoid insecticides are frequently employed in the cultivation and production of leafy vegetables. The conventional detection methods for these pesticides rely on chromatographic techniques, which are characterized by good precision and sensitivity. Nevertheless, these methods suffer from drawbacks such as complex sample pretreatment, prolonged detection times, and high costs, hindering the realization of on-site detection. This paper introduces a detection method based on surface-enhanced Raman spectroscopy (SERS) for the quantitative and qualitative analysis of pesticide residues in leafy vegetables. Gold nanoparticles (AuNPs) were meticulously synthesized to serve as the substrate for enhancing Raman signals. The average particle size was approximately 50 nm, and a significant absorption peak appeared at 536 nm. The density functional theory (DFT) with the B3LYP/6-311G was utilized to calculate the theoretical Raman spectra of the pesticides. The characteristic Raman peaks of the pesticides were selected as calibration peaks to establish calibration equations relating the concentration of pesticide residues to the intensity of these calibration peaks. By substituting the intensity of the calibration peak corresponding to the lowest detectable limit in the SERS spectra into the calibration equation, the quantitative detection limit was calculated. The study revealed that the detection limit for phosmet residues in Chinese cabbage could be was below 0.5 mg/kg, with an R^2^ of 0.93363, a standard deviation ranging from 3.87% to 8.56%, and recovery rates between 94.67% and 112.89%. For thiabendazole residues in water spinach, the detection limit could be below 1 mg/kg, with an R^2^ of 0.98291, a standard deviation of between 1.71% and 9.29%, and recovery rates ranging from 87.67% to 107.83%. In the case of acetamiprid residues in pakchoi, the detection limit could also be below 1 mg/kg, with an R^2^ of 0.95332, a standard deviation of between 4.00% and 9.10%, and recovery rates ranging from 90.67% to 113.75%. These findings demonstrate that the SERS-based detection method for the semi-quantitative and qualitative analysis of pesticide residues in leafy vegetables is an effective approach, enabling rapid and reliable detection of pesticide residues in leafy vegetables.

## 1. Introduction

Organophosphate, fungicidal, and neonicotinoid pesticides are commonly employed in the cultivation and production of agricultural products. These pesticides exhibit contact toxicity and stomach poisoning effects and possess weak systemic action, posing potential risks to the human immune system and central nervous system. They are widely used across various crops, including fruits and vegetables. To ensure the production of pollution-free and green vegetables, it is crucial to establish stringent pesticide residue standards. The Ministry of Agriculture has responded by formulating a series of such standards, such as NY/T761-2008 [1] and GB2763-2014 [2] and utilizing chromatographic techniques including liquid chromatography (LC), liquid chromatography–mass spectrometry (LC-MS), gas chromatography (GC), and gas chromatography–mass spectrometry (GC-MS) [3,4], These chromatographic detection methods are renowned for their high precision and sensitivity, making them indispensable tools for accurately quantifying trace levels of pesticide residues in agricultural products. Their widespread application underscores their importance in safeguarding food safety and promoting the production of green vegetables. However, despite their advantages, these chromatographic methods also suffer from notable drawbacks. They involve complex sample pretreatment procedures, lengthy detection times, and high costs, which hinder their suitability for real-time or on-site detection. Consequently, there is a pressing need to develop innovative detection methods that can overcome these limitations and provide more efficient, cost-effective, and user-friendly alternatives for pesticide residue analysis [5,6].

Raman spectroscopy is a commonly used technique for identifying small organic molecules, being especially suitable for pollutant detection [7,8]. Researchers have also discovered that pyridine molecules adsorbed onto gold or silver colloids could generate an exceptionally intense Raman spectrum. The intensity of this enhanced signal is highly dependent on the size of the gold or silver colloids and the excitation wavelength employed, but there is potential for a remarkable enhancement factor of 10^4^ to 10^6^ times greater than conventional Raman signals. This renowned phenomenon is known as the surface-enhanced Raman scattering (SERS) effect [9]. Owing to its ultra-high sensitivity, SERS technology has found widespread applications across various fields. It has been successfully utilized for in vitro and in vivo detection of diverse biomedical tissues [10,11], as well as for pesticide residue detection in fruits and vegetables [12]. Moreover, SERS has demonstrated its capability in single-molecule detection [13] and has made significant strides in detecting pesticide residues in food items such as grains, vegetables, and fruits [14,15]. For instance, it has been applied to detect pesticides like dimethoate, methamidophos, chlorpyrifos, acephate, and their metabolites, as well as combinations of pesticides such as methyl demeton, phosmet, and carbaryl, and even mixtures of multiple pesticides. Flexible 2D nanocellulose-based SERS substrate was used to detect pesticide residues in fruits. The substrate could sensitively detect dimethoate and acetamiprid residues on the surface of apples at 4.1 and 10.7 μg/L, respectively [16]. Mingchun Lv prepared a SERS substrate for pesticide residue detection on foods. It had greatly improved sensitivity, uniformity, and reproducibility, with a Raman enhancement factor of 3.01 × 10^7^. The limit of detection of malachite green was 0.13 ng/cm^2^ [17]. AuNPs for universal enhancement were prepared for SERS detection of pesticide residues. These had an ultrahigh sensitivity of 0.1 ppb for acetamiprid, paraquat, and carbendazim. And good reproductivity (RSD < 6%) and a strong linear relationship (R^2^ ≥ 0.97) were demonstrated for four pesticide residues through apple sample analysis. In summary, SERS (surface-enhanced Raman spectroscopy) technology has demonstrated the capability to detect pesticide residues in agricultural products, thereby fulfilling the demand for rapid and trace-level detection of such residues. Currently, there is a scarcity of literature reports on the non-destructive detection of organophosphate, fungicidal, and neonicotinoid pesticide residues in leafy vegetables using SERS technology.

In this study, leafy vegetables (Chinese cabbage, water spinach, and pakchoi) were selected as the test carriers, with organophosphate (phosmet), fungicidal (thiabendazole), and neonicotinoid (acetamiprid) pesticides as the target analytes. AuNPs were selected as the enhancement substrate. Rapid and non-destructive quantitative detection of pesticide residues in these leafy vegetables was conducted using SERS technology. The SERS spectra of pesticide residues in the leafy vegetables were acquired using a Raman spectrometer in conjunction with gold nanoparticles. The raw spectral data were subsequently preprocessed using preprocessing methods such as standard normal variate (SNV) transformation, normalization, and multiplicative scatter correction (MSC). The characteristic Raman peaks of the pesticides were selected as calibration peaks, and linear fitting of the data was performed using Origin software to establish calibration equations relating the concentration of pesticide residues to the intensity of these calibration peaks.

## 2. Materials and Methods

### 2.1. Experimental Instruments

Instruments included a portable Raman spectrometer (QE65pro, Ocean Optics Co., Ltd., Orlando, FL, USA), Raman microscopy platform (DAC151B785, NPL, Teddington, UK), high-precision electronic balance (EST200-4, Shenyang Tianping Instrument Co., Ltd., Shenyang, China), laboratory ultrapure water machine (P60-CY, Kertone Water Treatment Co., Ltd., Changsha, China), and vortex mixer (Vortex-Genie 2/2T, Shanghai Lingchu Environmental Protection Instrument Co., Ltd., Shanghai, China).

### 2.2. Experimental Samples and Reagents

Phosmet, thiabendazole, and acetamiprid of analytical grade were purchased from Shanghai Aladdin Reagent Co., Ltd. (Shanghai, China). Acetonitrile of analytical grade was purchased from Sinopharm Chemical Reagent Beijing Co., Ltd. (Beijing, China). Hydrogen tetrachloroaurate (III) trihydrate (HAuCl_4_·3H_2_O, ≥49.0%) was sourced from Sigma-Aldrich Trading Co., Ltd. (Shanghai, China). Sodium citrate tribasic and sodium chloride (all of analytical grade, ≥99%) were obtained from West Long Science & Technology Co., Ltd. (Guangzhou, China) Acetonitrile (CH_3_CN, chromatographic grade, ≥99.9%) was supplied by Shanghai Aladdin Bio-Chem Technology Co., Ltd. (Shanghai, China). The leafy vegetables (Chinese cabbage, water spinach, and pakchoi) were sourced from the Teaching and Experimental Base of Jiangxi Agricultural University.

### 2.3. Experimental Methods

#### 2.3.1. Gold Nanoparticle Preparation

To begin, all glassware was meticulously cleaned through immersion in aqua regia and subsequent rinsing for subsequent use. A 150 mL solution of 2.2 mM sodium citrate was then prepared in a three-necked round-bottom flask, followed by heating with continuous stirring for a duration of 15 min. Subsequently, 1 mL of a 25 mM HAuCl4 solution was introduced. After an additional 10 min of heating and stirring, the solution was gradually cooled to 90 °C, at which point 1 mL of a 60 mM sodium citrate solution was rapidly added. Following 2 min of vigorous stirring, an additional 1 mL of a 25 mM HAuCl_4_ solution was incorporated. The solution was consistently maintained at a constant temperature of 90 °C and stirred for a period of 30 min. At this juncture, 2 mL of the solution was extracted from the flask, and the cooling to 90 °C and subsequent reagent addition steps were repeated. Throughout this iterative process, the nanoparticle size of the gold nanoparticles progressively increased. This sequence of steps was repeated until the 14th iteration was achieved. Finally, the resultant gold colloid was allowed to cool to room temperature and then stored at 4 °C for subsequent utilization [18].

#### 2.3.2. Sample Preparation

Preparation of pesticide standard solutions: Accurately weigh 200 mg of each pesticide standard and place in a 200 mL volumetric flask. Dissolve each pesticide in acetonitrile to prepare a 1000 mg/L pesticide standard solution. Store the solutions at 4 °C in a dark environment. Acetamiprid is highly soluble in water, while phosmet has a solubility of 22 mg/L, making it slightly soluble in water. The solubility of thiabendazole is approximately 1 mg/L, making it poorly soluble in water. Further dilute the 1000 mg/L stock solutions with acetonitrile to prepare a series of standard working solutions with concentrations of 100, 50, 20, 15, 10, 5, 2, 1, 0.5, 0.4, 0.2, and 0.1 mg/L.

Simulation and extraction of pesticide residues in leafy vegetables: Wash and dry the fresh leafy vegetables. Accurately weigh the leafy vegetable samples to prepare 30 samples. The weights are as follows: Considering that the leafy vegetables are of different types and weights, with water spinach being the lightest and Chinese cabbage the heaviest for a given unit of size, different masses of the vegetables must be taken: 100 mg for water spinach, 200 mg for pakchoi, and 500 mg for Chinese cabbage. Apply different concentrations of the standard working solutions onto the surface of each leafy vegetable sample using a pipette, with 200 μL of pesticide standard working solution per sample. The concentration of the sample is approximately equal to the volume multiplied by concentrations of the standard working solutions and then divided by the mass of the leafy vegetable. Dry the samples at room temperature before taking SERS measurements. Considering the variations in concentration on different parts of the leafy vegetables, select three test points on each sample. Apply 100 μL of AuNPs onto each test point using a pipette gun and collect the SERS spectra of the samples. Take the average of the spectra obtained from the three test points.

#### 2.3.3. Raman Spectroscopy Data Collection

The Raman spectroscopy detection parameters are specified as follows: the excitation wavelength was set at 785 nm, with a laser power of 200 mW. The integration time for each measurement was 10 s, and the spectral resolution was 2 cm^−1^. Three scans were conducted and averaged for each sample. The scanning range spanned from 400 to 1800 cm^−1^. The procedure used was as follows: Place the solid pesticide standard onto a quartz plate. Use a coverslip to flatten the sample evenly. Employ the instrument’s built-in solid probe for direct detection of the sample. Utilize a pipette gun to apply 100 μL of AuNPs onto each of the three pre-selected test points on the sample surface for the collection of leafy vegetable samples. The SERS spectra are to be collected from these test points. Calculate the average spectrum from the three test points to represent the pesticide residue status of the sample [19]. The pure substrate spectrum, nanoparticle spectrum, acetonitrile spectrum, and pesticide SERS spectra were collected and compared to determine the SERS characteristic peaks of the pesticide.

#### 2.3.4. Computational Methodology

Density functional theory (DFT) represents a class of ab initio methods rooted in quantum mechanics and the Born–Oppenheimer approximation. It serves as a quantum chemical approach for investigating electronic structure theory, currently standing as a leading method in electronic structure calculations and one of the most frequently employed techniques in the field of computational chemistry [20]. In this study, the molecular configurations of pesticides were constructed using GaussView 3.07 software. Theoretical calculations were performed utilizing the Gaussian 03 quantum chemistry program package. Specifically, the B3LYP functional within the DFT framework was adopted, employing the 6-311G basis set. This basis set was applied uniformly to C, O, H, and N atoms. The outcomes of pesticide frequency calculations were visualized and analyzed using the GaussView 3.07 software platform. The “(d)” component signifies the inclusion of polarization functions for non-hydrogen atoms, which account for the electron density distortion caused by the presence of other atoms. The “(p)” indicates an addition set of polarization functions with *p*-type symmetry on hydrogen atoms. This computational approach enables a detailed examination of the electronic structure and vibrational properties of pesticide molecules, providing valuable insights into their chemical behavior and reactivity. There are some discrepancies between DFT and experimental results (solid Raman or SERS). The choice of functional and basis set may introduce discrepancies in the calculated frequencies. These differences arise primarily from enhancement mechanisms and molecular interaction. SERS relies on electromagnetic and chemical enhancement effects near noble metal nanostructures.

#### 2.3.5. Data Processing

To evaluate the sensitivity of the SERS method and establish the correlation between pesticide concentrations in leafy vegetable samples and the peak intensities of characteristic peaks in the collected SERS spectra, a series of SERS spectra were acquired from leafy vegetable samples with varying pesticide concentrations. The lowest concentration at which the characteristic pesticide peaks in the SERS spectra of leafy vegetable samples remained discernible was identified as the limit of detection (LOD), which is only used to evaluate the sensitivity of the analytical method, to determine whether pesticide residues can be detected, and for qualitative identification. To mitigate interference from baseline shifts, random noise, and background signals, three preprocessing methods—standard normal variate (SNV), normalization, and multiplicative scatter correction (MSC)—were applied to the raw spectral data. These preprocessing steps aimed to enhance the quality and reliability of the spectral data for subsequent analysis [21]. To further assess the sensitivity of the SERS method and quantify the relationship between pesticide residue concentrations in leafy vegetable samples and the peak intensities of characteristic peaks in the SERS spectra, SERS spectra were collected from samples with a range of concentrations. The characteristic Raman peaks of the pesticides were selected as the calibration peaks. Linear fitting of the data was performed using Origin software to establish a calibration equation relating pesticide concentrations to the intensities of the calibration peaks. The study also obtained the calibration curves based on other SERS peak intensity values and determined the selected correction curve by comparing higher R^2^ values. The intensity of the calibration peak corresponding to the LOD in the SERS spectrum was substituted into the calibration equation to calculate the limit of quantitation (LOQ), which represents the lowest concentration at which the pesticide can be reliably quantified. All data analyses were conducted using Microsoft Excel 2003, MATLAB R2010a, and OriginPro 8 software platforms [22].

## 3. Results and Discussion

### 3.1. Au Nanoparticle Characterization Analysis

**Figure 1:**
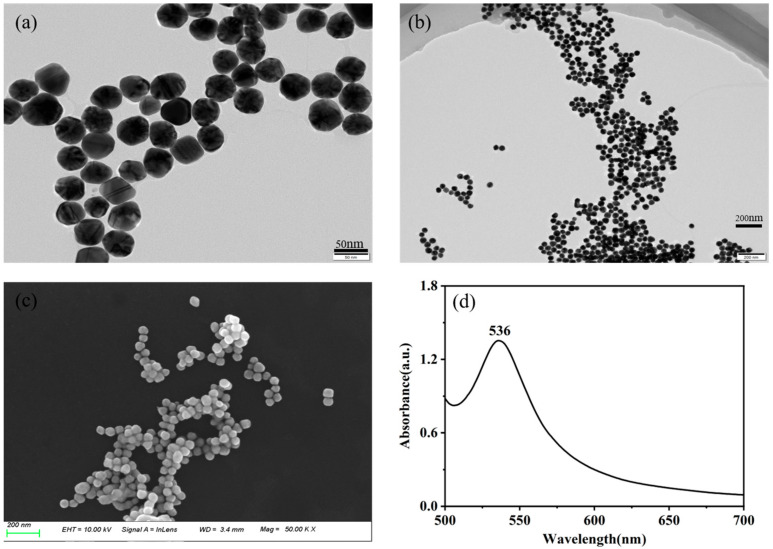
TEM image (**a**,**b**), SEM image (**c**), UV–Vis absorption spectra (**d**).

Figure 1 provides a comprehensive characterization of gold nanoparticles synthesized through a reduction method. As shown in the TEM images in Figure 1a,b, along with the SEM image in Figure 1c, gold nanoparticles are spherical and wine-red and exhibit slight sedimentation and aggregation over time. The average particle size was determined through calculations to be approximately 50 nm. In Figure 1d, the UV–Vis absorption spectrum reveals a prominent absorption peak at 536 nm, attributed to surface plasmon resonance (SPR), indicative of an estimated nanoparticle size of approximately 50 nm. These indicate that the prepared gold colloid is capable of providing a remarkable SERS enhancement.

### 3.2. Theoretical Calculations, Solid Powders, and SERS Spectra of Pesticides

**Figure 2:**
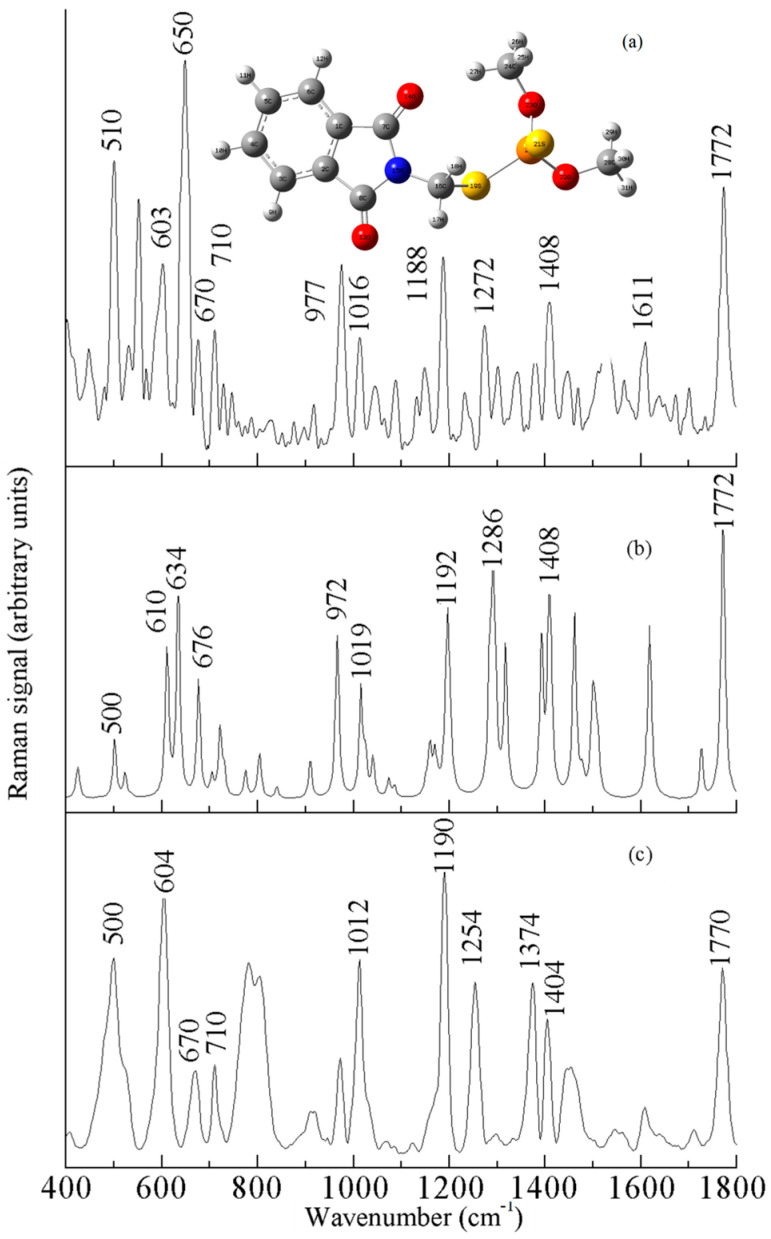
Raman spectrum of phosmet: (**a**) solid, (**b**) DFT-calculated, and (**c**) SERS.

The molecular formula of phosmet is C_11_H_12_NO_4_PS_2_, which contains a benzene ring and C-S, C=O, C-O, P=S, C-N, P-O-CH_3_, and C-H groups [23,24]. Figure 2a shows the experimentally measured Raman spectrum of phosmet powder, Figure 2b shows the theoretically calculated Raman spectrum of phosmet, and Figure 2c shows the surface-enhanced Raman spectrum of 10 mg/L phosmet. As can be seen in the figure, the theoretical Raman spectra and experimental Raman spectra were slightly different regarding peak intensities, but the same regarding the peak positions. The SERS spectra showed obvious peaks near 604 and 1190 cm^−1^, while the strongest peak of the theoretical spectra was at 1772 cm^−1^. The mutual force between molecules was not considered in the theoretical Raman spectra, and some peaks did not appear in the experimental Raman spectra. Comparison and analysis of Figure 2a–c provide a comprehensive fingerprinting of the vibrational modes of the Raman spectra of phosmet. In Figure 2c, there are five distinct Raman peaks located at 500, 604, 1012, 1190, and 1770 cm^−1^. These peaks are located at different positions from the acetonitrile SERS peaks of 380, 920, and 1370 cm^−1^. The Raman peak at 604 cm^−1^ is more pronounced and attributed to the stretching vibration of C-S with the in-plane deformation vibration of the C=O group. The Raman characteristic peak at 1190 cm^−1^ is caused by the out-of-plane deformation vibration of P-O-CH_3_. The 500 cm^−1^ peak was caused by the in-plane bending vibration of CH_2_ and PO_2_. The Raman peak at 1012 cm^−1^ can be attributed to the asymmetric deformation vibration of C-O, and the Raman peak at 1770 cm^−1^ is attributed to the stretching vibration of C=O. The other Raman peaks of phosmet is attributed as shown in Table 1.

**Figure 3:**
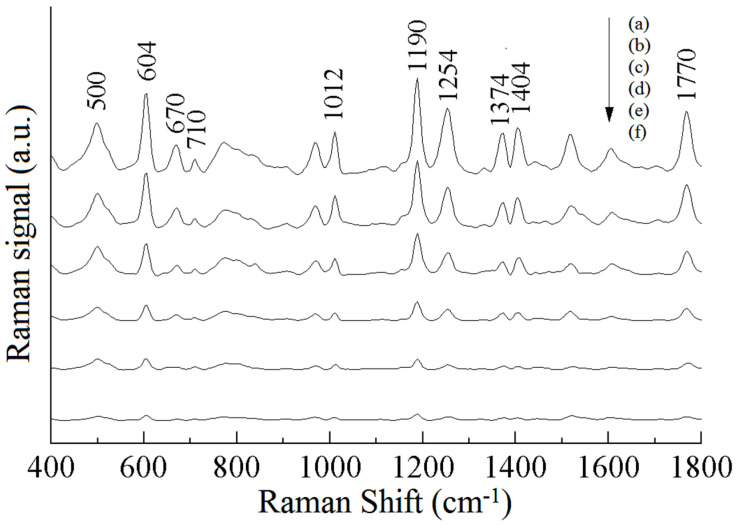
SERS spectra of phosmet with different concentrations, (a)–(f): 10, 5, 2, 1, 0.8, 0.5 mg/L.

Figure 3 shows the SERS spectra of the standard solutions of different concentrations of phosmet. As can be seen in Figure 3a, the Raman peaks at 500, 604, 710, 1012, 1190, 1254, 1404, and 1770 cm^−1^ are obvious. The Raman peak at 604 cm^−1^ is more obvious, which is attributed to the stretching vibration of C-S and accompanied by the in-plane deformation vibration of the C=O group; the Raman characteristic peak at 1190 cm^−1^ is caused by the out-of-plane deformation vibration of P-O-CH_3_; the Raman characteristic peak at 1770 cm^−1^ is attributed to the stretching vibration of C=O. It can also be seen from Figure 3 that the intensity of the Raman peaks of phosmet increases with increases in the concentration of the standard solution. The peaks at 500, 1012, 1254, 1404, and 1770 cm^−1^ change relatively fast, and those at 604 and 1190 cm^−1^ change slowly, and these Raman peaks can be used to analyze the pesticide residues of phosmet quantitatively. In Figure 3f, the Raman peaks at 604 and 1190 cm^−1^ can still be recognized, thus indicating that the detection of phosmet using SERS spectroscopy is feasible and able to reach a level of 0.5 mg/L or less.

**Figure 4:**
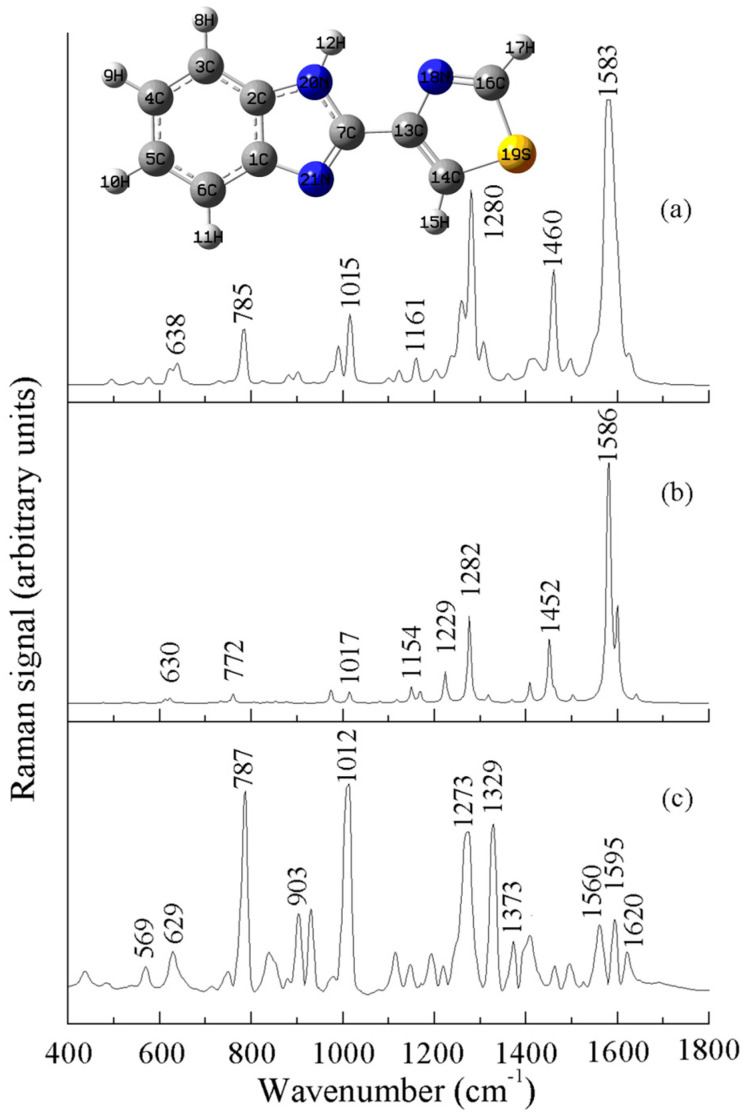
Raman spectrum of thiabendazole: (**a**) solid, (**b**) DFT-calculated, and (**c**) SERS.

The thiabendazole molecule contains a benzimidazole, a thiazole ring, and C=N, C-S-C, C-S, C=C, N-H, C-C, and C-H moieties. In Figure 4b, it can be seen that the thiabendazole molecule showed obvious Raman vibration peaks at 785, 1015, 1280, 1460, and 1583 cm^−1^, which can be attributed to the following five causes: the vibration peak at 1280 cm^−1^ was caused by the stretching vibration of the benzimidazole ring; the vibration peak at the position of 1460 cm^−1^ was caused by the anti-stretching vibration of the C-N-C group; the Raman peak at 1583 cm^−1^ is attributed to the stretching vibration of the benzimidazole ring; the Raman peak at 785 cm^−1^ is attributed to the anti-stretching vibration of C-S-C; and the Raman peak at 1015 cm^−1^ is attributed to the stretching vibration of C=C accompanied by the in-plane bending vibration of C-H [25,26]. The Raman characteristic peaks of thiabendazole and their attributions are shown in Table 2.

**Figure 5:**
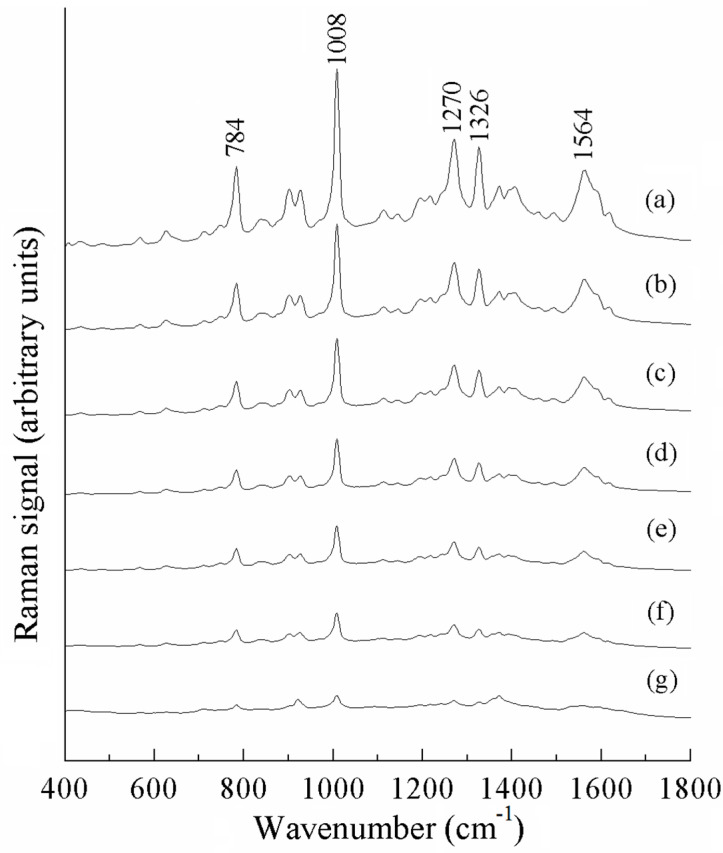
SERS spectra of thiabendazole solutions, (a)–(g): 10, 5, 2, 1, 0.8, 0.5, 0.2 mg/L.

Figure 5 shows the surface-enhanced Raman spectra of different concentrations of thiabendazole standard solutions. As can be seen in Figure 5a, the Raman peaks at 784, 1008, and 1270 cm^−1^ were obvious. These are located at different positions to the SERS characteristic peaks of acetonitrile solvent. The strongest Raman peak at 1008 cm^−1^ is attributed to the C=C stretching vibration and the breathing vibration of the benzimidazole ring, accompanied by the C-H in-plane bending vibration; the Raman peak at 1270 cm^−1^ is attributed to the stretching vibration of the thiazole ring, accompanied by the C-H in-plane bending vibration; and the Raman peak at 784 cm^−1^ is attributed to the C-H out-of-plane bending vibration. As can be seen in Figure 5, the intensity of the Raman peaks gradually decreased with decreases in thiabendazole concentration, and these Raman peaks can be used to quantitatively analyze thiabendazole pesticide residues. In Figure 5g, the Raman peaks at 784, 1008, and 1270 cm^−1^ can still recognized, thus indicating that the detection of thiabendazole using the SERS technique is feasible and able to reach a level of 0.2 mg/L or less.

**Figure 6:**
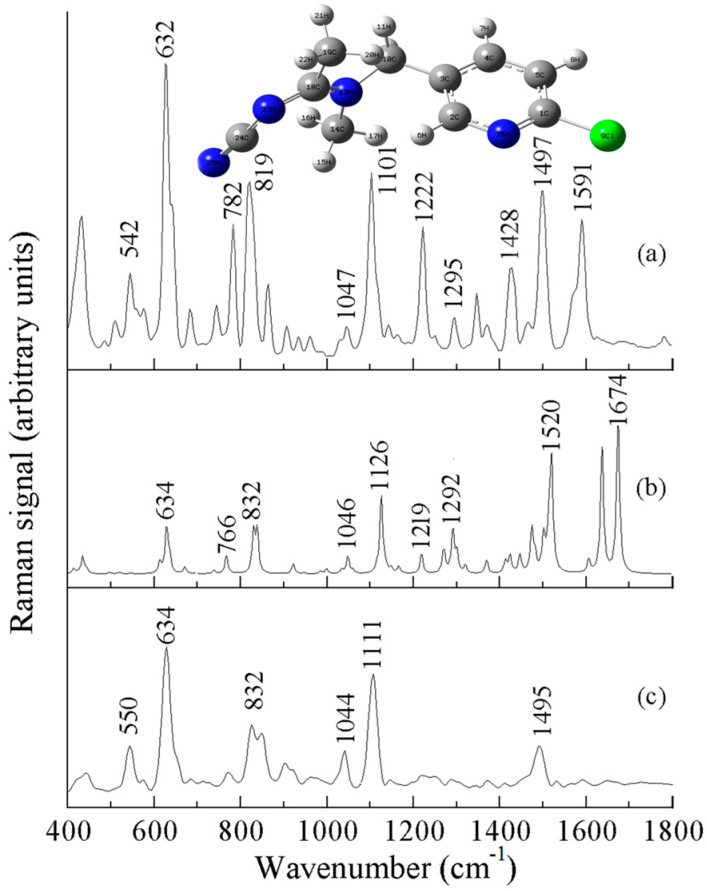
Raman spectrum of acetamiprid: (**a**) solid, (**b**) DFT-calculated, and (**c**) SERS.

Figure 6a is the experimentally measured Raman spectrum of acetamiprid powder, Figure 6b is the theoretically calculated Raman spectrum of acetamiprid, and Figure 6c is the experimentally obtained SERS of acetamiprid. As can be seen in Figure 6, the theoretical and experimental Raman spectra are slightly different. Some peaks in the theoretical Raman spectra did not appear in the experimental Raman spectra, and there are some differences in the peak intensities, but they are basically the same regarding the peak positions. The strongest peak in the experimental Raman spectrum and SERS spectrum appeared near 632 cm^−1^, while the strongest peak in the theoretical spectrum was at 1674 cm^−1^. Comparison and analysis of Figure 6a–c provide a comprehensive fingerprinting of the vibrational modes of the Raman spectra of acetamiprid, as shown in Table 3. Figure 6c shows the SERS spectra of 10 mg/L acetamiprid solution, with six distinct Raman peaks located at 550, 634, 832, 1044, 1111, and 1495 cm^−1^. The Raman peak at 634 cm^−1^ is more obvious and is attributed to the telescopic vibration of the C-Cl moiety. The characteristic Raman peak at 832 cm^−1^ was induced by the respiratory vibration of the benzene ring. The Raman peak at 1044 cm^−1^ was caused by the stretching vibration of the benzene ring. The Raman peak at 1111 cm^−1^ is attributed to the stretching vibration of the N-C=N group, and the Raman peak at 1495 cm^−1^ is attributed to the stretching vibration of the benzene ring [27,28].The Raman characteristic peaks of acetamiprid and their attributions are shown in Table 3.

**Figure 7:**
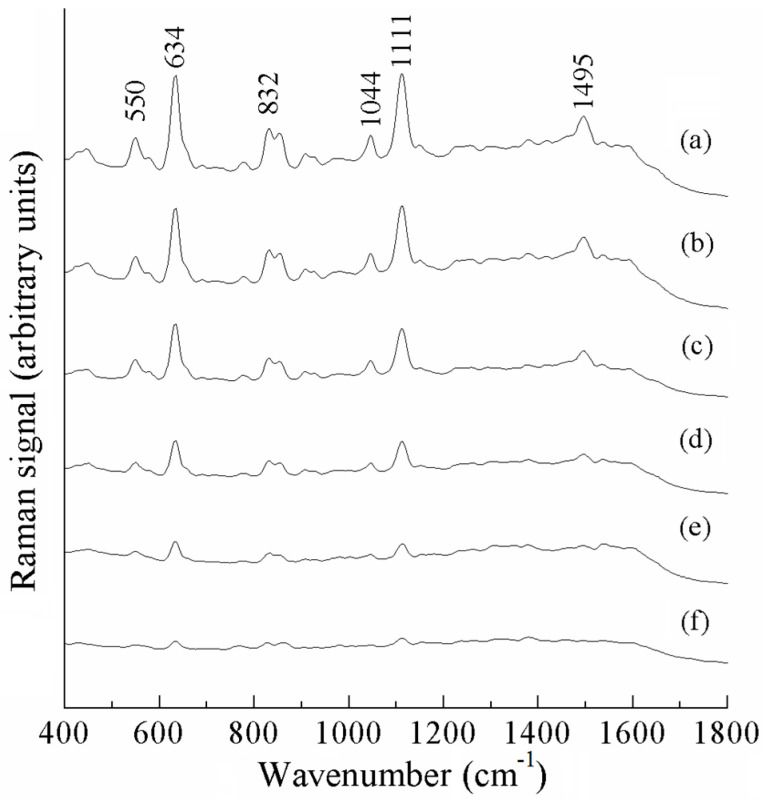
SERS spectra of acetamiprid solutions, (a)–(f): 10, 5, 2, 1, 0.8, 0.5 mg/L.

Figure 7 shows the surface-enhanced Raman spectra of different concentrations of acetamiprid standard solutions, from which it can be seen that the intensity of the Raman peaks of acetamiprid increases with increases in the concentration of the standard solution. The peaks at 550, 832, 1044, and 1495 cm^−1^ change relatively fast, and those at 634 and 1111 cm^−1^ change slower, which can be used to analyze the pesticide residues of acetamiprid quantitatively. These peaks are located at a different positions to the SERS characteristic peaks of acetonitrile solvent. The decrease in band intensity due to the decreasing concentration is not uniform across all bands because different bands are associated with distinct vibrational or electronic transitions of specific functional groups or molecular moieties in the sample. The sensitivity of these vibration modes to concentration changes may vary. In Figure 7f, the Raman peaks at 634 and 1111 cm^−1^ can still be recognized, thus indicating that the detection of acetamiprid using SERS is feasible and able to reach a level below 0.5 mg/L.

### 3.3. Detection of Pesticide Residues and Development of Calibration Curve

**Figure 8:**
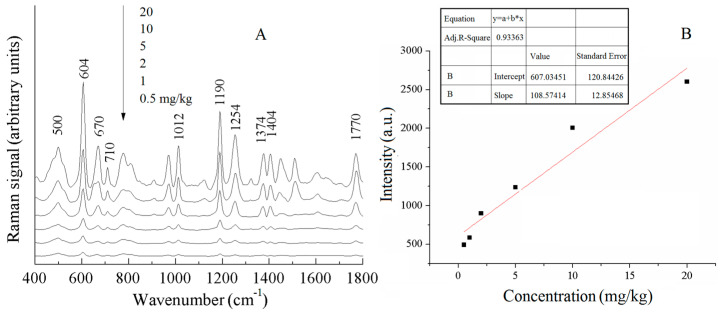
SERS spectra of phosmet pesticides in Chinese cabbage with different concentrations (**A**) and calibration curves (**B**).

Figure 8A shows the SERS spectra of different concentrations of phosmet pesticide residues in Chinese cabbage. The peak intensity of the phosmet characteristic peaks decreased with the concentration of pesticide residues, and the peaks at 604 and 1190 cm^−1^ were relatively weak when the concentration was 0.5 mg/kg, but they could be identified, meaning that the limit of detection of the phosmet pesticide in Chinese cabbage reached below 0.5 mg/kg. In Figure 8A, the intensity of the characteristic Raman peak of phosmet was enhanced with the increase in concentration, and after comparative analysis, the Raman peak at 604 cm^−1^ was finally selected as the calibration peak to develop the curve of the concentration of phosmet pesticide residues in Chinese cabbage and the intensity of the calibration peak. Figure 8B shows the calibration curves of different concentrations of phosmet pesticide residues in Chinese cabbage at 604 cm^−1^. The quantitative detection limit was calculated by substituting the lowest detection limit into the calibration equation.

**Figure 9:**
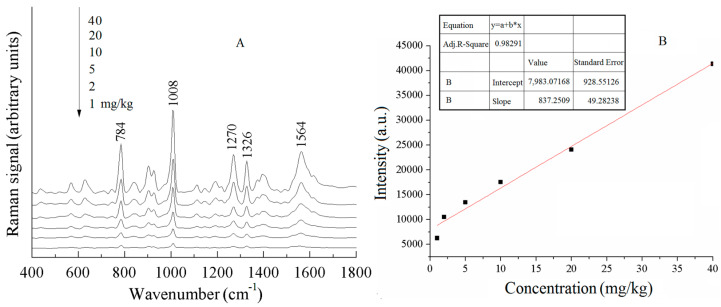
SERS spectra of thiabendazole pesticides in water spinach with different concentrations (**A**) and calibration curves (**B**).

Figure 9A shows the SERS spectra of different concentrations of thiabendazole pesticide residues in water spinach. The peak intensity of the thiabendazole characteristic peaks decreased with the decrease in the concentration of pesticide residues, and when the concentration was 1 mg/kg, the Raman peaks at 784, 1008, and 1270 cm^−1^ were relatively weak but still could be recognized, meaning that the limit of detection of the thiabendazole pesticide in water spinach reached below 1 mg/kg. In Figure 9A, the peaks at 784 and 1008 cm^−1^ were more obvious, and the intensity of Raman peaks at both 784 and 1008 cm^−1^ was enhanced with the increase in concentration. After comparative analyses, the Raman peak at 784 cm^−1^ was selected as the correction peak to develop the curves of the concentration of thiabendazole pesticide residues and the intensity of the correction peak. Figure 9B shows the calibration curves of different concentrations of thiabendazole pesticide residues at 784 cm^−1^ in water spinach, respectively.

**Figure 10:**
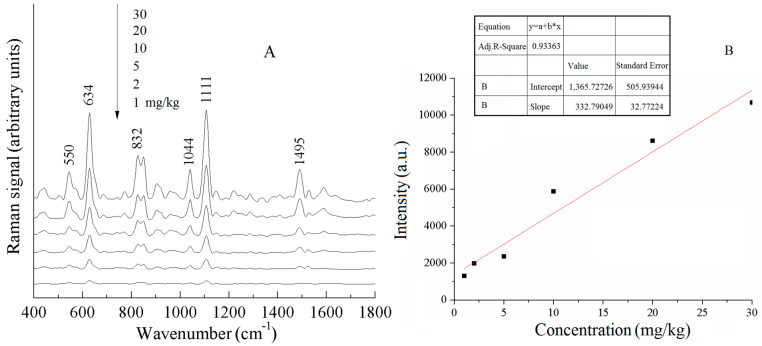
SERS spectra of acetamiprid pesticides in pakchoi with different concentrations (**A**) and calibration curves (**B**).

Figure 10A shows the SERS spectra of different concentrations of acetamiprid pesticide in Chinese cabbage. The peak intensity of the characteristic peaks of acetamiprid decreased with the decrease in the concentration of the pesticide residue, and when the concentration was 1 mg/kg, the Raman peaks at 634 and 1111 cm^−1^ were relatively weak, but they could still be recognized, meaning that the limit of detection of the acetamiprid pesticide in Chinese cabbage reached below 1 mg/kg. In Figure 10A, the intensities of the characteristic Raman peaks of acetamiprid were all enhanced with the increase in concentration, and after comparative analysis, the Raman peak at 1111 cm^−1^ was selected as the calibration peak to develop the curve of the concentration of acetamiprid pesticide residue in Chinese cabbage and the intensity of the surface-enhanced Raman signal. Figure 10B shows the calibration curve of different concentrations of acetamiprid pesticide residues in Chinese cabbage at 1111 cm^−1^.

The quantitative detection limit was calculated by substituting the lowest detection limit into the calibration equation. The calibration equation, correlation coefficient (R^2^), LOD, and LOQ of the curves are listed in Table 4. The calibration curves for the pesticide residues of phosmet, thiabendazole, and acetamiprid in leafy vegetables showed good linearity with correlation coefficients (R^2^) of 0.93363, 0.98291, and 0.95332, respectively, as can be seen in Table 4. As can be seen in Table 4, the calculated LOD and LOQ values were different but close, which further proves that the SERS method can be used to quantitatively analyze and detect the pesticide residues of phosmet, thiabendazole, and acetamiprid in leafy vegetables.

### 3.4. Accuracy Validation of the Model

To verify the accuracy of the method, the SERS signals were collected for five leafy vegetable samples with known concentrations, and the predicted values of the five leafy vegetable samples were obtained using the above method. Table 5 shows the results of the comparison between the added values and the predicted values of pesticide residues in leafy vegetables. The results of the comparison between the added values and the predicted values of phosmet pesticide in Chinese cabbage showed that the standard deviations ranged from 3.87% to 8.56%, and the recoveries ranged from 94.67% to 112.89%, which were basically the same between the predicted values and the added values. The results of the comparison between the added values and the predicted values of thiabendazole pesticide in water spinach showed that the standard deviations ranged from 1.71% to 9.29%, and the recoveries ranged from 87.67 to 107.83%. The results of comparing the added values with the predicted values of acetamiprid pesticide in pakchoi revealed that the standard deviations were 4.00–9.10%, and the recoveries were 90.67–113.75%, which demonstrated that the SERS method can be used to detect pesticide residues in leafy vegetables and the results were reliable.

## 4. Conclusions

The SERS technique was used to detect pesticide residues of phosmet, thiabendazole, and acetamiprid in leafy vegetables. The results showed that the SERS technique can achieve rapid detection of pesticide residues in leafy vegetables, and the whole process of the test, including sample and spectra acquisition, spectral analysis, and quantitative prediction, can be completed in 5 min, which is much lower than the time required by the traditional chromatographic detection method. The characteristic peaks of the three pesticides were relatively abundant, among which the wavenumbers of the characteristic peaks that could be used to identify the pesticide residues of phosmet were 500, 604, 710, 1012, 1190, 1254, 1404, and 1770 cm^−1^; the wavenumbers of the characteristic peaks of thiabendazole were 784, 1008, and 1270 cm^−1^; and the wavenumbers of the characteristic peaks of acetamiprid were 550, 634, 832, 1044, 1111, and 1495 cm^−1^. The Raman signals of phosmet at 604 cm^−1^, thiabendazole at 784 cm^−1^, and acetamiprid at 1111 cm^−1^ were selected as the correction peaks for the determination of pesticide residues in the samples. The prediction models were established respectively using calibration curves defined as y = 607 + 108x, y = 7983.1 + 837.2x and y = 1366 + 332.7x, which were allowed as a reference model for the quantitative analysis of pesticide residues of phosmet, thiabendazole, and acetamiprid in leafy vegetables. The results demonstrated that the SERS technique can achieve the rapid quantitative detection of pesticide residues of phosmet, thiabendazole, and acetamiprid in leafy vegetables, which provides a feasible solution for the rapid detection of other pesticide residues in food.

## Figures and Tables

**Table 1:** The Raman spectra (DFT-calculated, solid, and SERS) of phosmet and its assignments (cm^−1^).

^a^ DFT-Calculated	Solid	^b^ SERS	^c^ Assignment
500	510	500 (m)	ρ (CH2) or ρ (PO2)
610	603	604 (s)	ν (C− S), ρ (C=O)
634	650	−	τ (P=S)
676	670	670 (w)	τ (P=S)
−	710	710 (w)	benzene ring breathing
972	977	−	benzene ring breathing
1019	1016	1012 (m)	ν_as_ (C− O)
1192	1188	1190 (s)	ρ (P-O-CH_3_)
1286	1272	1254 (m)	ν (C− N)
1408	1408	1404 (m)	ρ (C− H)
1616	1611	−	ν (C=O)
1772	1772	1770 (m)	ν (C=O)

Notes: ^a^ B3LYP/6-31G, ^b^ s, strong; m, medium; w, weak, ^c^ ν, stretching; s, symmetric; τ, out-of-plane bending; as, asymmetric; ρ, in-plane deformation

**Table 2:** The Raman spectra (DFT, solid, and SERS) of thiabendazole and its assignments (cm^−1^).

^a^ DFT-Calculated	Solid	^b^ SERS	^c^ Assignment
-	-	569 (w)	ρ (C-C-C)
630	638	629 (m)	ρ (C-S-C)
772	785	784 (s)	ν_as_ (C-S-C)
-	-	903 (m)	ν (benzimidazole)
974	990	980 (w)	ν (C-S)
1017	1015	1008 (s)	ν (C=C), ρ (C-H)
1154	1161	1147 (w)	ρ (C-H) of benzimidazole
1282	1280	1270 (s)	ν (benzimidazole)
1326	1307	1329 (s)	ν (C=C)
1452	1460	1462 (w)	ν_as_ (C-N-C)
1586	1583	1560 (m)	ν (benzimidazole)
1606	1624	1595 (m)	ν (C=C), ν (C-C)

Notes: ^a^ B3LYP/6-31G, ^b^ s, strong; m, medium; w, weak, ^c^ ν, stretching; s, symmetric; out-of-plane bending; as, asymmetric; ρ, in-plane deformation

**Table 3:** The Raman spectra (DFT, solid, and SERS) of acetamiprid and its assignments (cm^−1^).

^a^ DFT-Calculated	Solid	^b^ SERS	^c^ Assignment
-	542	550 (m)	ν (Cl)
634	632	634 (s)	ν (C-Cl)
766	782	-	ν (C-Cl)
832	819	832 (m)	ring breathing vibration
1046	1047	1044 (w)	ν (ring)
1126	1101	1111 (s)	ν (N-C=N), ring breathing
1219	1222	-	ν (ring)
1292	1295	-	ρ (CH_2_)
1426	1428	-	ν (ring)
1520	1497	1495 (m)	ν (benzene ring)
1674		-	ν (C=C)

Notes: ^a^ B3LYP/6-31G, ^b^ s, strong; m, medium; w, weak, ^c^ ν, stretching; s, symmetric; out-of-plane bending; as, asymmetric; ρ, in-plane deformation

**Table 4:** The detection results of pesticide residues in leaf vegetables.

Calibration Band	Pesticides	Calibration Curves	*R* ^2^	LOD	LOQ
604 cm^−1^	phosmet	y = 607 + 108x	0.93363	0.5 mg/kg	0.76 mg/kg
784 cm^−1^	thiabendazole	y = 7983.1 + 837.2x	0.98291	1 mg/kg	1.17 mg/kg
1111 cm^−1^	acetamiprid	y = 1366 + 332.7x	0.95332	1 mg/kg	1.14 mg/kg

**Table 5:** Predicted value and added value of pesticide residues in leaf vegetables.

Pesticides	Sample	Added Value (mg/kg)	Predicted Value (mg/kg)	Standard Deviation (%)	Recovery (%)
phosmet	1	3	3.21	4.78	107.00
	2	6	6.57	6.41	109.50
	3	9	10.16	8.56	112.89
	4	12	11.36	3.87	94.67
	5	15	16.04	4.74	106.93
thiabendazole	1	3	2.63	9.29	87.67
	2	6	6.47	5.33	107.83
	3	9	9.22	1.71	102.44
	4	15	14.64	1.72	97.60
	5	30	31.93	4.41	106.43
acetamiprid	1	3	2.72	6.92	90.67
	2	6	5.67	4.00	94.50
	3	12	13.65	9.10	113.75
	4	18	19.62	6.09	109.00
	5	25	26.85	5.05	107.40

## Data Availability

The data collected in this research is available upon request.
